# 

**Published:** 1833-05

**Authors:** 


### MONTHLY LIST OF MEDICAL BOOKS.

[*Medical Works cannot be entered on this List unless a copy be sent for the purpose, the titles of Books having frequently been sent to us as published, which have not appeared for weeks, or even months, after.*]

A Plain Account of Vaccination, designed for the Heads of Families, wherein the History, Advantages, and Errors of this Subject are popularly treated. By A. B. C. pp. 58. London, Renshaw and Rush.

Part XV. (April.) The Cyclopædia of Practical Medicine. Edited by John Forbes, M.D., F.R.S., &c , Alex. Tweedie, M.D., &c., and John Conolly, M.D., &c. Containing *Menstruation* (concluded), by Dr. Locock; *Miliaria*, Dr. Tweedie; *Mortification*, Dr. Carswell; *Narcotics*, Dr. A. D. Thomson; *Nephralgia*, and *Nephritis*, Dr. H. W. Carter; *Neuralgia*, Dr. Elliotson; *Noli me Tangere*, or *Lupus*, Dr. Houghton; *Nyctalopia*, Dr. Grant; *Obesity*, Dr. Williams; *Œdema*, Dr. Derwall; *Ophthalmia*, Dr. Jacob; *Otalgia*, and *Otitis*, Dr. Burne; *Ovaria, Diseases of the*, Dr. Lee; *Palpitation*, Dr. Hope; *Pancreas, Diseases of*, Dr. Carter; *Paralysis*, Dr. R. B. Todd.—Large 8vo. pp. 137. Sherwood and Co., London.

Part XVII. The Principles and Practice of Obstetric Medicine, in a Series of Systematic Dissertations on Midwifery and on the Diseases of Women and Children. Illustrated by numerous Plates. By D. D. Davis, M.D., M.R.S.L., Professor of Midwifery in the University of London, &c.—4to. John Taylor, London.

A Treatise on the Venereal Disease, and its Varieties. By Wm. Wallace, M.R.I.A. &c., Surgeon to the Jervis-street Infirmary, Dublin.—8vo. pp. 382. Burgess and Hill, London.

Observations on Impediments of Speech, with some Remarks on their successful Treatment; in a Letter addressed to T. J. Pettigrew, Esq. F.R.S. &c. By Richard Cull.—8vo. pp. 32.—Renshaw and Rush, London.

The Black Death in the Fourteenth Century; from the German of J. F. C. Hecker, M.D. &c. Translated by B. G. Babington, M.D.—12mo. pp. 205. Schloss, London.

Succinct Practical Observations on the Effects of Bloodletting; containing an Investigation of the Practice of General and Local Abstraction of Blood; also how far Leeches may be efficacious, independently of the Evacuations they produce. To which are added, Observations on Visceral Inflammation after Parturition. By E. Geoghegan, M.R.C.S.I. &c.—8vo. pp. 60. Longman, London.

**A List of Persons who have obtained Certificates of their Fitness and Qualifications to practise as Apothecaries, from August 1st, 1815, to July 31, 1832; agreeably to the Act 55 Geo. III.—London.**

**Regulations to be observed by Students intending to qualify themselves to practise as Apothecaries in England and Wales. 1832.**

---

### APOTHECARIES' HALL.

**Names of Gentlemen to each of whom the Court of Examiners have granted Certificates:**

MARCH 28.

Frederick Bastone, of Minehead
James Burrow, Manchester
Clark Duchesne, London
Mark Brown Garrett, Shaftsbury
Henry Robert Jenkins, Peterborough
Robert Poyntz M'Donald, Bristol
John Crook Rumsey, Beaconsfield
Patrick Reilly
Wm. Kenrick Watson, Stourport

APRIL 4.

Samuel Sanders, of London
John Storrar
Wm. John Stephens Tuckwell, Burford

APRIL 9.

Wm. John Craken Grierson
Samuel La Mert, Spitalfields
David Thomas Morton, Rotherhithe

APRIL 18.

Thomas Bartlett, Devonport
Cuthbert Collingwood Embleton, Durham
Henry Tracey, Stonehouse

APRIL 25.

Henry Amos Ash, Bristol
Thomas Weston, Lambeth
Thomas Christopher Wood.

---

